# Association Between Dietary Fiber Intake and Inflammatory Biomarkers in U.S. Adults: A Cross-Sectional Analysis of the Pre-COVID-19 National Health and Nutrition Examination Survey 2017–2018

**DOI:** 10.3390/nu18060972

**Published:** 2026-03-19

**Authors:** Pablo Albiña-Palmarola, Yella Rottländer, Aracelly Solís Moyano, Hans Henkes

**Affiliations:** 1 Neuroradiologische Klinik, Kopf- und Neurozentrum, Klinikum Stuttgart, 70174 Stuttgart, Germany; hhhenkes@aol.com; 2 Klinik für Nieren-, Hochdruck- und Autoimmunerkrankungen, Klinikum Stuttgart, 70174 Stuttgart, Germany; y.rottlaender@klinikum-stuttgart.de; 3 Abteilung für Physiotherapie, Robert-Bosch-Krankenhaus Lungenzentrum Stuttgart, 70376 Stuttgart, Germany; asolis.moyano@gmail.com; 4 Medizinische Fakultät, Universität Duisburg-Essen, 47057 Essen, Germany

**Keywords:** dietary fiber, high-sensitivity C-reactive protein (hs-CRP), national health and nutrition examination survey (NHANES), systemic inflammation

## Abstract

Background/Objectives: Dietary fiber has been associated with lower levels of inflammatory biomarkers, but nationally representative evidence using recent U.S. data remains limited. We evaluated the association between dietary fiber intake and inflammatory biomarkers in U.S. adults using the National Health and Nutrition Examination Survey (NHANES) 2017–2018, the last fully completed cycle before the COVID-19 pandemic, providing a pre-pandemic benchmark for future comparisons. Methods: We analyzed 3570 adults (≥20 years) from NHANES 2017–2018 with complete dietary and biomarker data. Fiber intake was averaged from two 24 h recalls. Outcomes included serum high-sensitivity C-reactive protein (hs-CRP; primary outcome), white blood cell count (WBC), and neutrophil count. Survey-weighted regression models adjusted for demographic, socioeconomic, lifestyle, clinical, and dietary covariates. Associations were examined continuously (per 5 g/day fiber), by quartiles, and with restricted cubic splines. Sensitivity analyses excluded participants with cardiometabolic conditions or modified covariate sets. Results: Each 5 g/day higher fiber intake was associated with 4–7% lower hs-CRP (*p* < 0.001). Participants in the highest versus lowest fiber quartile had 20.7% lower hs-CRP (95% CI −27.1, −14.4) and 47% lower odds of elevated hs-CRP (OR 0.53, 95% CI 0.32–0.85). Secondary outcomes showed significant inverse associations: each +5 g/day was associated with −0.98% WBC (95% CI −1.84, −0.13; *p* = 0.024) and −1.44% neutrophils (95% CI −2.62, −0.26; *p* = 0.017) in fully adjusted models. Spline analyses showed no nonlinearity for WBC (*p* = 0.227) but nonlinear inverse associations for neutrophils (*p* = 0.0017). Sensitivity analyses confirmed robustness to exclusion of individuals with diabetes, hypertension, or hyperlipidemia, and to alternative covariate specifications. Conclusions: Higher dietary fiber intake was independently associated with a more favorable inflammatory biomarker profile (hs-CRP, WBC, and neutrophils) in U.S. adults, providing a pre-pandemic benchmark for future comparisons. Longitudinal and interventional studies are needed to clarify temporality and causality.

## 1. Introduction

Systemic low-grade inflammation plays a critical role in the development and progression of chronic diseases, including cardiovascular disease, diabetes, obesity, and metabolic syndrome. Among circulating inflammatory biomarkers, high-sensitivity C-reactive protein (hs-CRP) is widely recognized as a sensitive and reliable marker of systemic inflammation and an established predictor of cardiovascular risk. The American Heart Association (AHA) and Centers for Disease Control and Prevention (CDC) categorize individuals with hs-CRP levels > 3 mg/L as being at high cardiovascular risk, citing their two-fold increased risk compared to those with baseline hs-CRP < 1 mg/L [1]. Elevated hs-CRP levels, even within the subclinical range below 3 mg/L, have been associated with adverse health outcomes across diverse populations [2,3,4,5].

Dietary factors are increasingly recognized as key modulators of systemic inflammation. In particular, dietary fiber, a non-digestible carbohydrate obtained primarily from plant-based foods, has demonstrated anti-inflammatory and immunomodulatory properties through multiple mechanisms. Experimental and epidemiological studies suggest that higher fiber intake reduces systemic inflammation by improving gut microbiota composition, promoting the production of short-chain fatty acids (SCFAs), and regulating body weight and metabolic health. SCFAs, particularly butyrate, acetate, and propionate, modulate immune cell function through G-protein-coupled receptor signaling and histone deacetylase inhibition, resulting in anti-inflammatory effects both locally and systemically [6,7,8,9,10].

However, despite growing evidence supporting fiber’s anti-inflammatory effects, findings across studies examining the specific relationship between dietary fiber intake and hs-CRP remain inconsistent. While some large-scale studies using NHANES data have demonstrated inverse associations between fiber intake and hs-CRP levels, others have found stronger associations with alternative inflammatory markers such as interleukin-6 and tumor necrosis factor-α (TNF-α) receptor 2 (TNF-R2) rather than hs-CRP specifically. The relationship between fiber intake and inflammatory markers in large, nationally representative samples thus remains incompletely defined, particularly regarding hs-CRP as the primary outcome measure [11,12]. Recent meta-analyses and umbrella reviews underscore this complexity. Dietary fiber interventions appear to reduce some inflammatory markers more consistently than CRP in adults, whereas broader dietary patterns rich in fiber—particularly Mediterranean and vegetarian patterns—show more consistent inverse associations with CRP and related biomarkers in observational research [13,14,15]. These findings suggest that the relationship between fiber intake and inflammation may depend on both the biomarker studied and the overall dietary context. Beyond cardiometabolic risk, CRP is embedded in rheumatology treat-to-target strategies through composite indices such as the 28-joint Disease Activity Score using CRP (DAS28-CRP). Thus, population evidence linking higher dietary fiber intake to lower hs-CRP has clinical relevance for immune-mediated arthritides [16]. SCFAs may act via histone deacetylase inhibition and G-protein–coupled receptors to reinforce epithelial barrier integrity, expand regulatory T cells, and constrain Th17-skewed inflammation—axes central to rheumatoid arthritis (RA) pathophysiology [17,18,19].

The National Health and Nutrition Examination Survey (NHANES) provides a unique opportunity to investigate this association, given its robust sampling methodology, standardized dietary assessment protocols using the Automated Multiple-Pass Method for 24 h recalls, and availability of biomarker data. The NHANES dietary assessment methodology has been validated as the gold standard for quantifying actual dietary intakes in large population studies, with trained staff obtaining recalls using standardized protocols to minimize measurement error. While several recent analyses using NHANES have explored dietary fiber in relation to immune and metabolic health outcomes, focused evaluations of fiber intake and hs-CRP-defined systemic inflammation remain limited, particularly in the most recent survey cycles [2,20]. Furthermore, methodological challenges—including changes in survey protocols [21,22] and pandemic-related disruptions [23]—underscore the need for a single-cycle analysis to avoid systematic bias and ensure comparability of biomarker and dietary data. Understanding this relationship using pre-pandemic data is particularly important given that the COVID-19 pandemic significantly altered dietary patterns, inflammation levels, and health behaviors [24,25], making the 2017–2018 cycle crucial for establishing baseline associations in normal conditions.

Therefore, the present cross-sectional study aimed to examine the association between dietary fiber intake and the odds of elevated hs-CRP among adults in the pre-COVID-19 NHANES 2017–2018 cycle. We hypothesized that higher dietary fiber intake would be inversely associated with the prevalence of elevated hs-CRP (defined as >3 mg/L), independent of demographic, socioeconomic, and lifestyle factors, based on established mechanisms linking fiber to anti-inflammatory effects through gut microbiota modulation and SCFA production.

## 2. Materials and Methods

### 2.1. Study Population

We analyzed data from the 2017–2018 cycle of the U.S. National Health and Nutrition Examination Survey (NHANES), a cross-sectional survey conducted by the National Center for Health Statistics using a complex, multistage probability sampling design. All participants provided written informed consent, and the NHANES protocol was approved by the National Center for Health Statistics Ethics Review Board. The NHANES 2017–2018 public-use files were accessed on 9 September 2025, and the authors did not have access to information that could identify individual participants at any time. We focused specifically on the NHANES 2017–2018 cycle for several methodological reasons: (1) to avoid systematic bias from combining survey cycles with different methodologies, laboratory protocols, and population weighting schemes [22,26]; (2) to utilize the most recent NHANES cycle fully completed before COVID-19 pandemic disruptions; and (3) to establish baseline fiber-inflammation associations before pandemic-related changes in dietary behaviors, physical activity patterns, and psychological stress that could confound these relationships [24,25]. This study is reported in accordance with the STROBE statement for cross-sectional studies.

**Figure 1:**
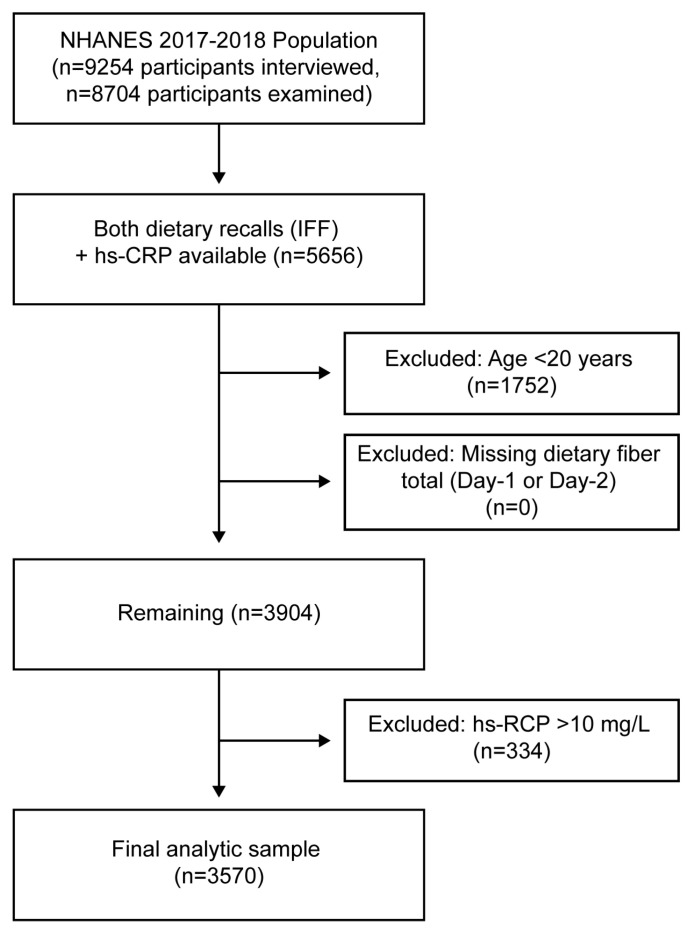
Participant flow diagram for analytic sample selection. Starting from the 2017–2018 NHANES examined sample (n = 9254), participants were sequentially excluded based on missing dietary recall data, absence of hs-CRP values, age < 20 years, and hs-CRP > 10 mg/L (to minimize influence of acute inflammation). The final analytic cohort included 3570 adults with complete data on both dietary recalls, dietary fiber intake, and hs-CRP within the analytic range. hs-CRP, high-sensitivity C-reactive protein; IFF, Individual Foods File; NHANES, National Health and Nutrition Examination Survey.

We included adults aged ≥ 20 years with complete data from both 24 h dietary recalls and measured serum high-sensitivity C-reactive protein (hs-CRP). Participants with hs-CRP > 10 mg/L were excluded to reduce potential bias from acute infections or major inflammatory states. After applying these criteria, the final analytic sample consisted of 3570 participants, representing approximately 188 million U.S. adults after applying survey weights. A flow diagram of sample selection is presented in Figure 1.

### 2.2. Exposure: Dietary Fiber Intake

Dietary intake was assessed by two interviewer-administered 24 h dietary recalls (Day 1 in person, Day 2 by telephone) using the U.S. Department of Agriculture Automated Multiple Pass Method. Dietary fiber intake (g/day) was calculated using the Food and Nutrient Database for Dietary Studies. We calculated the mean intake across the two recalls as the primary exposure. Fiber intake was analyzed as a continuous variable (per 5 g/day increment), in quartiles, and using restricted cubic splines (RCS) with knots at the weighted 5th, 35th, 65th, and 95th percentiles. Total energy intake (kcal/day) was calculated as the mean of Day 1 and Day 2 recalls and included as an adjustment variable.

### 2.3. Outcomes

The primary outcome was serum hs-CRP (mg/L), modeled both as a continuous variable (log-transformed to normalize distribution) and dichotomized at ≥3 mg/L vs. <3 mg/L, following AHA/CDC guidelines for cardiovascular risk classification [1]. Secondary outcomes were white blood cell count (WBC, ×10^3^/µL) and neutrophil count (×10^3^/µL), both modeled as continuous variables on the natural log scale. All biomarkers were assayed in NHANES laboratories following standardized protocols with quality assurance procedures.

### 2.4. Covariates

Potential confounders were selected a priori based on prior literature and a directed acyclic graph (Supplementary Figure S1). All models adjusted for age, sex, race/ethnicity, and poverty–income ratio. Lifestyle factors included smoking status (ever/never), vigorous physical activity (yes/no), and alcohol intake. Alcohol consumption was derived from the NHANES Alcohol Use Questionnaire and expressed in grams of ethanol per day. For the main models, alcohol was categorized into none, light–moderate (>0–14 g/day for women; >0–28 g/day for men), or heavy (>14 g/day for women; >28 g/day for men), consistent with U.S. dietary guidelines. In sensitivity analyses, alcohol intake was modeled continuously (grams/day). Clinical covariates included body mass index (BMI, kg/m^2^), self-reported diabetes, hypertension, and hyperlipidemia. Hyperlipidemia was defined as a composite variable incorporating physician diagnosis, use of lipid-lowering medication, or abnormal lipid levels (total cholesterol, LDL, HDL, or triglycerides). Total energy intake (kcal/day) was included as a dietary covariate. Other health conditions (e.g., asthma, cancer, chronic kidney disease) and selected medication groups (NSAIDs, corticosteroids, and disease-modifying antirheumatic drugs (DMARDs)/biologics) were summarized in descriptive analyses but not included in the primary regression models, as they were not identified as confounders in the directed acyclic graph (Figure S1). In addition, these medications are often prescribed in response to inflammatory or chronic conditions that may themselves influence hs-CRP, so routine adjustment could introduce confounding by indication or over-adjustment rather than improve causal interpretation. Detailed definitions and coding procedures for alcohol intake, hyperlipidemia, kidney function, central adiposity, and medication use are provided in Supplementary Methods and are summarized in Supplementary Table S1.

### 2.5. Statistical Analysis

All analyses accounted for the NHANES complex survey design using sampling weights, strata, and primary sampling units, with two-day dietary recall weights (WTDR2D) applied. We specified NHANES design variables (SDMVPSU and SDMVSTRA) and applied the 2-day dietary sample weights because the exposure was defined as the mean of Day 1 and Day 2 recalls. This approach yields nationally representative estimates for participants with complete two-day dietary data. Baseline characteristics were described across quartiles of dietary fiber intake using weighted means or proportions, with linear or Rao–Scott χ^2^ tests for trend. For primary analyses, we fitted survey-weighted multivariable linear regression models to examine the association between fiber intake and log-transformed hs-CRP, WBC, and neutrophil counts. Survey-weighted logistic regression was used to estimate odds ratios (ORs) and 95% confidence intervals (CIs) for elevated hs-CRP (≥3 mg/L). Fiber was modeled continuously (per 5 g/day), by quartiles, and using RCS to assess potential nonlinear relationships. Nonlinearity was tested with Wald tests for spline terms beyond the first. Predicted probabilities and adjusted geometric means across the 5–35 g/day intake range were estimated and visualized with 95% CIs. The derived analytic dataset and reproducibility code are available in a public repository [27].

### 2.6. Sensitivity Analyses

To assess robustness, we conducted the following sensitivity analyses:

1. Restriction to a healthier subset excluding individuals with diabetes, hypertension, hyperlipidemia, or cardiovascular disease, to minimize reverse causation.
2. Exclusion of adiposity adjustment, omitting BMI from models to evaluate its role as a potential mediator rather than a confounder.
3. Alternative modeling of alcohol intake, using continuous grams/day (S3A) rather than categorical definitions.
4. Exclusion of heavy alcohol consumers (S3B) to test whether extreme alcohol use biased results.

### 2.7. Software

All analyses were performed with Stata version 17.0 (StataCorp, College Station, TX, USA). Two-sided *p*-values < 0.05 were considered statistically significant.

## 3. Results

### 3.1. Study Sample

A total of 9254 participants were initially examined in NHANES 2017–2018. After excluding individuals < 20 years of age, those missing dietary recall data, or without hs-CRP measurements, 3570 adults remained in the analytic sample (Figure 1). The distribution of hs-CRP values in this population is shown in Figure S2. Serum hs-CRP was right-skewed, with most values < 3 mg/L. Overall, 27.3% of participants had hs-CRP concentrations ≥ 3 mg/L, while 5.8% had concentrations > 10 mg/L and were excluded from analyses.

### 3.2. Baseline Characteristics

Weighted baseline characteristics are presented in Table 1 (stratified by hs-CRP categories). The analytic cohort had a mean age of 47.1 years, 51.2% were women, and 62.8% were non-Hispanic White. Median dietary fiber intake was 16.2 g/day (IQR 11.1–23.4 g/day). Participants with elevated hs-CRP (≥3 mg/L) were more likely to be women, non-Hispanic Black, to have higher BMI, hypertension, diabetes, and hyperlipidemia, and reported lower socioeconomic status and physical activity. By contrast, the composite variable “any chronic condition” did not differ significantly between hs-CRP groups (*p* = 0.39), likely reflecting the heterogeneity of the conditions grouped within this summary category.

### 3.3. Unadjusted Associations

Scatter plots of dietary fiber intake against inflammatory biomarkers are provided in Figure S3. In unadjusted survey-weighted regressions, dietary fiber was inversely associated with hs-CRP, WBC, and neutrophil count.

### 3.4. Multivariable Associations with hs-CRP

**Figure 2:**
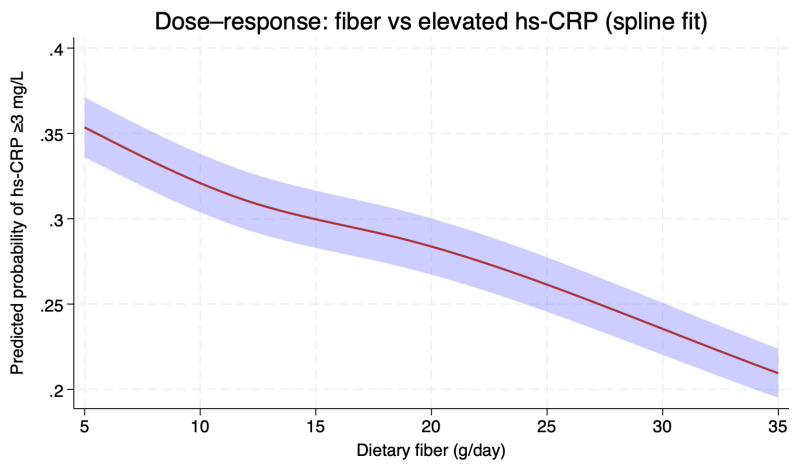
Dose–response association between dietary fiber intake and the predicted probability of elevated hs-CRP (≥3.0 mg/L) among U.S. adults, NHANES 2017–2018. Curves were derived from survey-weighted logistic regression using restricted cubic splines with knots at the 5th, 35th, 65th, and 95th percentiles of fiber intake. The model was fully adjusted for age, sex, race/ethnicity, family income-to-poverty ratio, body mass index, smoking, vigorous physical activity, self-reported diabetes, hypertension, hyperlipidemia, total energy intake, and alcohol use. The shaded region represents 95% confidence intervals.

In survey-weighted linear models adjusted for demographics (Model 1), each 5 g/day higher dietary fiber intake was associated with 6.99% lower hs-CRP (95% CI −8.61, −5.38; *p* < 0.001). After further adjustment for socioeconomic status, BMI, smoking, and physical activity (Model 2), the association attenuated but remained significant (−4.67%, 95% CI −6.11, −3.22; *p* < 0.001). Adding clinical factors (self-reported diabetes, hypertension, and hyperlipidemia), total energy intake, and alcohol (Model 3) yielded a similar estimate (−4.53%, 95% CI −6.32, −2.74; *p* < 0.001). Consistent findings were observed when modeling hs-CRP as binary (3.0–9.9 mg/L): each 5 g/day higher fiber was associated with lower odds of elevated hs-CRP (Model 1 OR ≈ 0.86, *p* < 0.001; Model 2 OR 0.88 [~0.81–0.94], *p* < 0.001; Model 3 OR ~0.87, *p* < 0.01). Table 2 summarizes these results, and Figure 2 depicts the adjusted dose–response using RCS.

### 3.5. Quartile Analyses

When dietary fiber intake was modeled in quartiles, participants in the highest quartile (Q4) had significantly lower inflammation compared with those in the lowest quartile (Q1). In fully adjusted models, Q4 was associated with a 20.7% lower mean hs-CRP (95% CI −27.1 to −14.4; *p* < 0.001). Intermediate quartiles (Q2 and Q3) were not statistically different from Q1. A test for linear trend across quartiles, treating quartile as an ordinal variable, showed a significant dose–response pattern (−7.0% per quartile, 95% CI −10.5 to −3.6; *p* = 0.001). For the binary outcome (hs-CRP 3.0–9.9 mg/L), the highest quartile had 47% lower odds of elevated hs-CRP compared with the lowest quartile (OR 0.53, 95% CI 0.32–0.85; *p* = 0.012). A significant trend across quartiles was observed (OR per quartile 0.83, 95% CI 0.70–0.98; *p* = 0.032). Pairwise contrasts confirmed that Q4 differed significantly from both Q2 and Q3 (all *p* ≤ 0.02), whereas Q2 and Q3 did not differ from each other (Table 3).

### 3.6. Secondary Outcomes

Higher fiber intake was inversely associated with leukocyte indices (Table 4). In fully adjusted models (Model 3), each 5 g/day increase in fiber was associated with 0.98% lower WBC (95% CI −1.84, −0.13; *p* = 0.024) and 1.44% lower neutrophils (95% CI −2.62, −0.26; *p* = 0.017). Results were stronger in models without clinical covariates and attenuated with additional adjustment, but directions were consistent across specifications.

### 3.7. Dose–Response Analysis

To explore the functional form of the association, we fitted RCS for dietary fiber intake (knots at the 5th, 35th, 65th, and 95th percentiles). The spline model confirmed a graded inverse association between fiber intake and the probability of elevated hs-CRP (≥3.0 mg/L). The curve was approximately linear across the observed range (*p* for nonlinearity = 0.94). Predicted probabilities of elevated hs-CRP decreased steadily from ~35% at 5 g/day to ~22% at 35 g/day of fiber intake (Figure 2). In survey-weighted restricted cubic spline models, higher fiber intake was associated with lower adjusted geometric mean of WBC, with no evidence of nonlinearity (Wald test of spline terms beyond the linear component *p* = 0.227). Across the prespecified range (5 to 35 g/day), the adjusted geometric mean WBC decreased from approximately 7.26 ×10^9^/L to 6.73 × 10^9^/L (≈7% lower at 35 vs. 5 g/day). For neutrophils, the association was nonlinear (*p* = 0.0017), characterized by a steeper decline at lower intakes and a relative plateau at higher intakes. Over 5 to 35 g/day, the adjusted geometric mean neutrophil count decreased from about 4.31 × 10^9^/L to 3.77 × 10^9^/L (≈13% lower at 35 vs. 5 g/day) (Figure S4).

### 3.8. Sensitivity Analyses

In sensitivity analyses, results were generally consistent with the main models (Table 5). Restricting the analysis to 640 participants free of diabetes, hypertension, hyperlipidemia, and cardiovascular disease (S1) yielded attenuated estimates, likely due to reduced sample size. Each additional 5 g/day of fiber intake was associated with −4.62% lower hs-CRP (95% CI −9.94%, +0.70%; *p* = 0.089), with an OR of 0.91 for elevated hs-CRP (95% CI 0.70, 1.16; *p* = 0.41). WBC (−0.97%, 95% CI −2.11%, +0.17%) and neutrophil counts (−1.71%, 95% CI −3.75%, +0.32%) were directionally consistent but not statistically significant. Excluding BMI and waist circumference from the models (S2) strengthened the inverse associations, suggesting partial mediation by adiposity. Fiber intake was associated with −6.26% lower hs-CRP per +5 g/day (95% CI −8.15%, −4.37%; *p* < 0.001), and the OR for elevated hs-CRP was 0.85 (95% CI 0.80, 0.91; *p* < 0.001). Associations with WBC (−1.24%, 95% CI −2.08%, −0.41%; *p* = 0.004) and neutrophils (−1.78%, 95% CI −2.93%, −0.63%; *p* = 0.002) became statistically significant in this model. Replacing alcohol categories with continuous grams/day (S3A) produced estimates nearly identical to the primary analyses. Each 5 g/day increase in fiber was associated with −6.43% lower hs-CRP (95% CI −9.27%, −3.59%; *p* < 0.001), with an OR of 0.82 for elevated hs-CRP (95% CI 0.73, 0.90; *p* < 0.001). In contrast, WBC (−0.99%, 95% CI −2.24%, +0.26%) and neutrophil counts (−1.03%, 95% CI −2.68%, +0.62%) showed no significant associations. Finally, excluding heavy drinkers (S3B) did not materially alter results. Fiber intake was associated with −5.63% lower hs-CRP per +5 g/day (95% CI −8.62%, −2.63%; *p* < 0.001), with an OR of 0.83 for elevated hs-CRP (95% CI 0.74, 0.93; *p* = 0.004). WBC (−0.90%, 95% CI −2.35%, +0.54%) and neutrophil counts (−0.66%, 95% CI −2.62%, +1.30%) remained non-significant in this restricted population.

## 4. Discussion

This nationally representative analysis of U.S. adults found that higher dietary fiber intake was independently associated with lower levels of inflammatory biomarkers. Each 5 g/day increment in fiber intake was associated with 4–7% lower serum hs-CRP, and participants in the highest fiber quartile had 21% lower mean hs-CRP and 47% lower odds of elevated hs-CRP (≥3 mg/L) than those in the lowest quartile. Dose–response analysis showed a largely linear inverse association across the typical range of fiber intakes, with no evidence of threshold effects or nonlinearity for hs-CRP. Secondary outcomes showed modest inverse associations with WBC and a significant nonlinear association with neutrophils. Together, these findings add to the observational evidence linking dietary fiber intake with a more favorable inflammatory biomarker profile in the contemporary U.S. population.

### 4.1. Biological Plausibility

Multiple complementary mechanisms support these findings. Fermentable fibers promote the growth of saccharolytic gut microbiota and the production of SCFAs, such as butyrate, which have anti-inflammatory and immunomodulatory effects [28,29]. SCFAs directly modulate immune responses by modulating T cell differentiation, reducing pro-inflammatory cytokines such as IL-17, and enhancing regulatory T cell populations through hydroxycarboxylic acid receptor 2 (HCA2)-dependent mechanisms [30]. Additionally, SCFAs enhance epithelial barrier integrity, limit systemic endotoxemia, and inhibit histone deacetylase activity affecting gene transcription [31]. These metabolites activate GPR43, GPR41, and GPR109A receptors, promoting immune homeostasis and reducing inflammatory cytokine production. Fiber intake also improves insulin sensitivity, reduces visceral adiposity, and enhances satiety, which may indirectly attenuate chronic low-grade inflammation [32]. Our analyses showed that BMI adjustment attenuated but did not eliminate fiber-hs-CRP associations, suggesting adiposity explains part but not all observed effects. Furthermore, the nonlinearity detected for neutrophils suggests that fiber may influence leukocyte dynamics through pathways beyond adiposity, possibly via modulation of gut–immune signaling [33]. Our inverse fiber–hs-CRP association aligns with data that SCFAs bolster barrier integrity and restrain IL-6/IL-17/TNF-α signaling, which underlies synovitis (RA) and enthesitis (spondyloarthritis), plausibly lowering hs-CRP [34,35,36].

### 4.2. Comparison with Previous Literature

Our findings align with previous epidemiological research, though important methodological distinctions exist across study designs that affect comparability and interpretation of results.

#### 4.2.1. Longitudinal Cohort Studies

Prospective cohort studies provide the strongest observational evidence for fiber’s anti-inflammatory effects due to their ability to establish temporal relationships and minimize reverse causation. The Cardiovascular Health Study of 4125 adults aged 65+ found that a 5 g/d increase in total fiber was associated with significantly lower CRP and IL-1 receptor antagonist concentrations over time. Notably, only cereal fiber was consistently associated with lower inflammation across multiple markers, and inflammation mediated 16.1% of the association between cereal fiber and cardiovascular disease, providing mechanistic insight into fiber’s cardioprotective effects [37]. This source-specific finding highlights an important research question that our total fiber analysis cannot address. While our results show significant inverse associations between total dietary fiber intake and inflammatory biomarkers, future research should examine whether specific fiber sources drive these effects. A Massachusetts longitudinal study of 524 subjects [38] found an inverse association between total dietary fiber intake (16.11 g/d average) and CRP concentrations in both cross-sectional and longitudinal analyses with quarterly follow-up visits over one year. Participants in the highest quartile of fiber intake had a 63% lower likelihood of elevated CRP, which is consistent with our findings of 47% lower odds. Large prospective cohorts have provided additional evidence supporting fiber’s anti-inflammatory effects, though with important nuances regarding fiber subtypes. In the Nurses’ Health Study [39], cereal fiber intake was inversely associated with CRP and TNF-R2 among 902 diabetic women, with CRP levels 18% lower in the highest versus lowest quintile of cereal fiber intake, while fruit and vegetable fiber showed no significant associations. Additionally, the Multi-Ethnic Study of Atherosclerosis found that dietary patterns high in whole grains and fruit were inversely associated with CRP, IL-6, and homocysteine [40], though this study examined dietary patterns rather than isolated fiber intake, limiting direct comparisons with our results.

#### 4.2.2. Cross-Sectional Studies

Single-cycle NHANES analyses demonstrate remarkable consistency with our results. Ajani et al. [41], using NHANES 1999–2000 data from 3920 participants, found that participants in the highest quintile of total fiber intake (32 g/d) had 51% lower odds of elevated CRP compared to the lowest quintile (5.1 g/d) (OR: 0.49; 95% CI: 0.37, 0.65). This finding aligns closely with our quartile analysis showing 47% lower odds for elevated hs-CRP in the highest versus lowest fiber quartile. The remarkable consistency of effect sizes across nearly two decades suggests that the fiber-inflammation relationship has remained stable in the U.S. population despite changing dietary patterns and food environments. King et al. [42], also using NHANES 1999–2000 data from 4900 participants, demonstrated an independent inverse association between dietary fiber intake and CRP levels after adjusting for multiple confounders. Their findings provided early evidence for the fiber-inflammation relationship using the same single-cycle approach we employed. The fact that similar associations persist across different analytical approaches and covariate adjustments strengthens confidence in the underlying biological relationship.

#### 4.2.3. Multi-Cycle Studies: Methodological Considerations

Several recent studies combining multiple NHANES cycles have reported findings generally consistent with ours. Multi-cycle analyses are a standard and informative approach in NHANES research when survey weights, design features, and cycle-specific methodological differences are handled appropriately, and they offer the important advantage of larger sample sizes and greater statistical power. Qi et al. [43], combining NHANES 2015–2020 data from 14,392 participants, found that dietary fiber intake was inversely associated with systemic immune-inflammation index, neutrophil-to-lymphocyte ratio, and hs-CRP. Similarly, Randall et al. [44], using NHANES 2015–2018 data, reported inverse associations between dietary fiber and hs-CRP concentrations. At the same time, pooling NHANES cycles requires attention to several sources of methodological heterogeneity that may affect comparability and interpretation. These include cycle-to-cycle changes in survey methodology, questionnaire content, and laboratory procedures; updates in the Food and Nutrient Database for Dietary Studies, with revised food codes and nutrient calculations across survey cycles; and differences in weighting frameworks across NHANES periods [22,26,45,46]. For analyses spanning 2015–2020, additional complexity arises from the COVID-19 disruption of the 2019–March 2020 cycle, which required special primary sampling unit reassignments and weighting adjustments in the pre-pandemic file [21,26]. We therefore selected the single 2017–2018 cycle to provide a clear pre-pandemic benchmark within one internally consistent survey cycle, rather than to imply that pooled multi-cycle analyses are inherently invalid.

#### 4.2.4. Intervention Evidence and Meta-Analytic Synthesis

Limited intervention evidence exists in general populations for fiber’s effects on inflammatory markers, with most controlled trials conducted in special populations with unique inflammatory profiles. Xie et al. [47] found that fermentable dietary fiber supplementation (10–20 g/day for 6 weeks) significantly decreased CRP, IL-6, IL-8, and TNF-α levels in hemodialysis patients. However, these findings have limited generalizability to healthy populations due to the unique inflammatory profile and metabolic dysfunction characteristic of end-stage renal disease. The substantial baseline inflammation in dialysis patients may make anti-inflammatory interventions more readily detectable than in healthy populations with lower baseline CRP levels.

Meta-analytic findings vary by population and study design. A comprehensive umbrella review of 52 meta-analyses involving 47,197 subjects found that dietary fiber interventions significantly reduced TNF-α levels but showed no statistically significant effect on CRP across 7 meta-analyses, including 2780 adult participants from randomized controlled trials (RCTs) [13]. This finding highlights important heterogeneity in fiber’s effects across different inflammatory pathways and suggests that hs-CRP may be less responsive to fiber interventions than other inflammatory markers in controlled trial settings. A meta-analysis of 10 RCTs in children and adolescents found significant CRP reductions following dietary fiber interventions, with fiber supplementation proving more effective than fiber-rich foods [14]. Notably, this pediatric meta-analysis found no significant effects for IL-6 or TNF-α, which contrasts with the adult evidence, where TNF-α showed greater responsiveness than CRP. These contrasting findings suggest age-related differences in inflammatory responses to fiber intake, potentially reflecting varying baseline inflammation levels, metabolic profiles, or gut microbiome maturation across different life stages.

The recent umbrella review by Reyneke et al. (2025) searched five major databases without age restrictions and incorporated 30 systematic reviews covering 225 unique primary studies—158 intervention trials (65% randomized controlled) and 67 observational studies—constituting the most comprehensive synthesis of dietary pattern effects on inflammation to date [15]. In this analysis, the Mediterranean dietary pattern—inherently high in fiber through its emphasis on whole grains, fruits, vegetables, and legumes—demonstrated the strongest evidence for anti-inflammatory effects. Five out of eight meta-analyses of intervention trials reported significant CRP reductions ranging from −0.37 to −1.04 mg/L, with 67% of intervention trials and 80% of observational studies showing beneficial effects on inflammatory markers. Vegetarian dietary patterns showed significant inverse associations with CRP in observational studies (−0.61 to −3.91 mg/mL range), though RCTs found no effect, highlighting the consistent pattern of stronger observational versus interventional evidence. These complementary analyses highlight three key insights: isolated fiber interventions in healthy adults yield modest CRP effects but improve other markers such as TNF-α; pediatric populations appear more responsive to fiber for reducing CRP than adults; and fiber consumed within broader dietary patterns demonstrates stronger anti-inflammatory associations than when administered as a standalone supplement. The null findings for CRP in adult intervention meta-analyses, in contrast to our positive observational results, suggest that population-based observational associations may reflect broader dietary patterns and lifestyle factors that controlled supplementation trials cannot capture. Our NHANES analysis captures total fiber within real-world dietary contexts and baseline inflammation levels that may be more conducive to detecting fiber-inflammation associations than controlled trials in healthy volunteers. Moreover, the umbrella review’s finding that fiber-rich dietary patterns ameliorate low-grade inflammation in adults with chronic conditions underscores the clinical relevance of our results in a representative U.S. population.

### 4.3. Rationale for Selecting NHANES 2017–2018

Our decision to focus specifically on the NHANES 2017–2018 cycle was motivated by several methodological considerations. As already mentioned, this approach was chosen to maximize internal consistency and avoid the added complexity of combining survey cycles with evolving survey methodology, laboratory protocols, and weighting schemes [21,22,26]. More importantly, the 2017–2018 cycle represents the most recent complete, nationally representative dietary and biomarker data available before the COVID-19 pandemic fundamentally disrupted survey operations [48]. The pandemic led to unprecedented changes in NHANES methodology starting with the 2019–2020 cycle, which was suspended in March 2020 and could not produce nationally representative estimates [23]. The subsequent 2021–2022 cycle involved major methodological changes, including telephone interviews instead of in-person interviews, significant content reduction, and modified examination procedures. These pandemic-driven changes make recent cycles potentially incomparable with historical data and unsuitable for establishing baseline pre-pandemic associations. Moreover, avoiding the post-COVID era is particularly important for dietary and inflammation research, as the pandemic significantly altered dietary behaviors, physical activity patterns, healthcare access, and psychological stress levels—all factors that could confound the fiber-inflammation relationship [24,25]. The 2017–2018 cycle thus represents the last “normal” survey cycle before these unprecedented disruptions.

### 4.4. Clinical and Public Health Implications

Our findings suggest that higher dietary fiber intake is associated with a more favorable inflammatory profile at the population level. The largely linear association across a broad intake range indicates that even modest differences in fiber intake are associated with lower hs-CRP. Given the persistently low fiber consumption in the U.S.—averaging approximately 16 g/day in our sample, well below the recommended 21–38 g/day [49]—these findings remain relevant from a public health perspective. However, the observed associations were modest and should not be interpreted causally. The 47% lower odds of elevated hs-CRP (≥3 mg/L) in the highest versus lowest fiber quartile may be meaningful at a population level, but residual confounding and reverse causation cannot be excluded. At the individual level, these associations should be interpreted as modest rather than dramatic. However, even modest shifts in inflammatory biomarker distributions may be relevant from a public health perspective when fiber intake is low across a large proportion of the population. This interpretation is also consistent with the broader literature, in which observational studies and dietary-pattern analyses often show stronger associations than isolated fiber supplementation trials. Accordingly, our results are best interpreted as supporting current dietary guidance and motivating further longitudinal and interventional work, rather than demonstrating that increasing fiber intake directly reduces inflammation or cardiovascular risk [50]. The dose–response relationship indicates public health recommendations should emphasize not just achieving minimum fiber intake levels, but maximizing intake within safe ranges. Although our analysis targets population inflammation rather than disease activity, randomized and observational studies suggest that higher-fiber, Mediterranean-style eating can improve RA disease activity (DAS28-CRP) and associate with lower RA risk—a clinical context that is concordant with our inverse fiber–hs-CRP findings [51,52]. In addition, recent large datasets also link cereal fiber with lower RA prevalence [53].

### 4.5. Study Strengths and Limitations

Several features enhance confidence in our findings. The use of NHANES 2017–2018 data allows generalization to the U.S. adult population, owing to the nationally representative sampling strategy. Additionally, dietary intake was assessed by two 24 h recalls using the validated Automated Multiple-Pass Method, which provides greater precision than a single recall and represents the gold standard for dietary assessment in population studies [54]. By applying complex survey weights and design variables, we minimized bias from unequal probabilities of selection and nonresponse. Additionally, we conducted extensive sensitivity analyses, including alternative modeling of alcohol intake, exclusion of heavy drinkers and individuals with cardiometabolic diseases, and omission of BMI, all of which yielded consistent results. Finally, the use of RCS provided reassurance that associations were not driven by outliers or nonlinear intake patterns.

Nevertheless, important limitations must be acknowledged. The cross-sectional design precludes establishing temporality and does not allow causal inference; reverse causation cannot be ruled out, as individuals with inflammation-related comorbidities or broader health concerns may have changed their diet before assessment. Although our sensitivity analyses restricting to healthier participants yielded directionally consistent results, they do not eliminate reverse causation or residual confounding from unmeasured health behaviors, and statistical power was reduced in that subset. Our analysis examined total dietary fiber intake without distinguishing between fiber sources (cereal, fruit, vegetable). While this approach aligns with current dietary guidelines that emphasize total fiber intake [49], it prevents assessment of whether specific fiber types drive the observed anti-inflammatory associations. Previous research suggests differential effects by fiber source, with cereal fiber showing more consistent anti-inflammatory associations than fruit or vegetable fiber [37,39]. This limitation restricts our ability to contribute to the mechanistic understanding of fiber’s anti-inflammatory effects and limits the specificity of dietary recommendations that can be derived from our findings. Future analyses should examine fiber intake by source to identify optimal dietary strategies for inflammation reduction. In addition, higher fiber intake may reflect broader differences in overall diet quality rather than an isolated nutrient effect. Residual confounding by other dietary features—such as saturated fat intake, refined carbohydrate intake, or overall dietary pattern quality—cannot be excluded and may partly contribute to the observed associations. Similarly, although anti-inflammatory medications were described and operationalized in the Supplementary Materials, they were not included in the primary models because of their likely position downstream of inflammatory conditions and the risk of confounding by indication. Residual confounding related to medication use therefore cannot be fully excluded. Measurement error is another concern: 24 h dietary recalls are subject to recall bias, and two days of recall may not fully capture usual long-term dietary intake. Day-to-day variability may therefore attenuate associations and contribute to misclassification of habitual fiber intake [55,56]. Archer et al. [57] have raised concerns about the validity of NHANES dietary data, reporting that energy intake estimates may be physiologically implausible in a substantial proportion of participants, though their critique primarily focused on energy underreporting rather than fiber intake specifically. hs-CRP variability represents an additional limitation. Ockene et al. [58] and more recently Gough et al. [59] have documented considerable within-subject variation in hs-CRP measurements (coefficient of variation 0.41–0.44), with only modest agreement between serial measurements when classified by quartile or clinical cutpoints. This could attenuate observed associations and necessitates caution in interpreting single hs-CRP measurements. Residual confounding from unmeasured behaviors (e.g., supplement use, unrecorded medication use, over-the-counter non-aspirin nonsteroidal anti-inflammatory drugs) or socioeconomic determinants cannot be excluded. Additionally, while hs-CRP is a sensitive marker of systemic inflammation, it is influenced by transient conditions, and although we excluded values >10 mg/L, subclinical infections could still bias estimates. Finally, the exclusion of younger participants (<20 years) limits applicability to adolescents.

### 4.6. Directions for Future Research

Future research should prioritize longitudinal analyses using prospective cohort designs to strengthen causal inference and address temporal relationships, while intervention trials should identify specific fiber types most effective for inflammation reduction, building on evidence suggesting differential effects of cereal versus fruit/vegetable fiber sources [37]. Within NHANES 2017–2018, an important next step would be to use the Individual Foods Files to decompose total fiber intake into food-source-specific categories such as cereal/grain, fruit, vegetable, and legume-derived fiber. Although feasible, this approach requires food-level linkage across both dietary recalls, prespecified classification rules for mixed dishes and multi-ingredient foods, and careful harmonization of source categories, which was beyond the scope of the present total-fiber analysis. Such analyses may help clarify whether the inverse associations observed here are driven preferentially by specific fiber sources and whether source-specific patterns differ in their biological relevance. Mechanistic studies integrating microbiome and metabolomic data are needed to clarify the relative contributions of SCFA production, gut barrier function, and metabolic improvements to fiber’s anti-inflammatory effects [60]. Research should examine whether fiber’s effects vary by individual characteristics such as baseline microbiome composition, genetic polymorphisms affecting fiber metabolism, or presence of metabolic dysfunction. Finally, given methodological challenges in dietary assessment, future studies should explore objective biomarkers of fiber intake, longer-term dietary monitoring using mobile technology, or integration of multiple assessment methods to reduce measurement error [61].

## 5. Conclusions

Higher dietary fiber intake was independently associated with lower hs-CRP concentrations and lower odds of elevated inflammation in U.S. adults, with complementary findings from WBC and neutrophil analyses. These results are consistent with current dietary guidance and support the relevance of dietary fiber as a marker of a healthier inflammatory profile, but they should not be interpreted as causal. Longitudinal and interventional studies are needed to clarify temporality, residual confounding, and the extent to which increasing dietary fiber intake may influence inflammatory biomarkers. Focusing on the NHANES 2017–2018 cycle provides a clear pre-pandemic benchmark for future comparisons.

## Figures and Tables

**Table 1:** Characteristics of the analytic cohort (NHANES 2017–2018), by hs-CRP category.

Characteristic	<3 mg/L (n = 2432)	3–10 mg/L (n = 1138)	*p*-Value
**Continuous variables, mean (95% CI)**			
Age, years	48.0 (46.2–49.7)	49.0 (47.4–50.6)	0.33
PIR	3.22 (3.05–3.38)	2.99 (2.84–3.13)	**0.03**
Total energy intake, kcal/day	2124 (2060–2187)	1999 (1926–2072)	**0.01**
Dietary fiber intake, g/day	17.5 (16.6–18.5)	15.1 (14.2–15.9)	**<0.001**
BMI, kg/m^2^	27.9 (27.3–28.4)	32.9 (32.2–33.7)	**<0.001**
Waist circumference, cm	96.7 (95.0–98.3)	108.4 (106.6–110.1)	**<0.001**
eGFR, mL/min/1.73 m^2^	95.7 (93.4–97.9)	95.1 (93.7–96.5)	0.61
WBC, ×10^3^/µL	6.94 (6.69–7.19)	8.05 (7.83–8.26)	**<0.001**
Neutrophils, ×10^3^/µL	3.95 (3.81–4.09)	4.79 (4.64–4.93)	**<0.001**
**Categorical variables, % (95% CI)**			
Female	47.9 (44.2–51.7)	57.9 (51.2–64.4)	**0.02**
Race/Ethnicity			**0.03**
– Mexican American	8.7 (5.5–13.3)	9.1 (6.2–13.3)	
– Other Hispanic	6.1 (4.6–8.1)	8.8 (6.6–11.5)	
– Non-Hispanic White	64.4 (57.4–70.8)	60.6 (52.5–68.2)	
– Non-Hispanic Black	10.1 (7.5–13.4)	12.4 (8.1–18.6)	
– Non-Hispanic Asian	6.8 (4.9–9.2)	3.9 (2.7–5.6)	
– Other/Multiracial	3.9 (3.1–5.0)	5.2 (3.1–8.4)	
Education			**0.03**
– ≤High school	34.7 (29.8–40.0)	40.7 (34.9–46.9)	
– Some college/AA	29.6 (25.5–34.1)	33.5 (28.9–38.4)	
– College graduate+	35.7 (29.2–42.8)	25.8 (19.9–32.7)	
Smoking (≥100 cigarettes, lifetime)	38.9 (35.2–42.7)	45.9 (41.1–50.8)	**0.02**
Physical activity (vigorous)	34.1 (29.5–39.1)	23.7 (19.6–28.3)	**0.001**
Alcohol consumption (sex-specific)			0.12
– None	8.1 (6.2–10.6)	11.2 (8.3–14.9)	
– Light–Moderate	50.1 (46.8–53.5)	44.9 (39.4–50.5)	
– Heavy	41.8 (37.7–46.0)	43.9 (39.1–48.9)	
Hypertension	29.1 (25.0–33.5)	38.6 (33.7–43.7)	**0.002**
Diabetes	9.5 (7.9–11.3)	14.8 (12.1–17.8)	**0.004**
Hyperlipidemia	65.4 (61.3–69.3)	80.9 (75.5–85.4)	**<0.001**
Central obesity	52.0 (47.3–56.7)	80.5 (76.2–84.3)	**<0.001**
CKD (eGFR < 60)	5.7 (4.5–7.2)	6.6 (5.0–8.5)	0.35
Arthritis	26.5 (22.8–30.5)	30.8 (25.3–37.0)	0.08
Asthma	12.8 (10.9–14.9)	15.6 (12.5–19.3)	0.14
COPD	3.9 (2.9–5.2)	4.8 (3.0–7.4)	0.32
CVD	7.9 (6.4–9.6)	10.9 (7.6–15.4)	0.07
Cancer history	9.7 (7.6–12.2)	11.7 (9.3–14.5)	0.33
Any chronic condition	43.5 (39.7–47.3)	44.9 (40.6–49.5)	0.39
NSAIDs	5.7 (4.4–7.3)	8.5 (5.7–12.5)	0.08
Corticosteroids	2.0 (1.3–2.9)	1.6 (1.1–2.4)	0.48
DMARDs	1.0 (0.5–2.1)	1.0 (0.6–1.7)	0.97

Values are weighted means with 95% confidence intervals for continuous variables and weighted proportions with 95% confidence intervals for categorical variables, incorporating NHANES sampling weights, strata, and PSUs. *p*-values are derived from adjusted Wald tests (continuous variables) or Rao–Scott χ^2^ tests (categorical variables). Smoking reflects ≥100 cigarettes in lifetime (SMQ020). Vigorous physical activity reflects PAQ605 (yes/no). Alcohol categories are sex-specific. Sample sizes may vary across characteristics due to item-level missingness; estimates use all available observations for each variable under the NHANES complex survey design. Abbreviations: BMI, body mass index; CKD, chronic kidney disease; COPD, chronic obstructive pulmonary disease; CVD, cardiovascular disease; DMARDs, disease-modifying antirheumatic drugs; eGFR, estimated glomerular filtration rate; hs-CRP, high-sensitivity C-reactive protein; NHANES, National Health and Nutrition Examination Survey; PIR, poverty–income ratio; WBC, white blood cell count.

**Table 2:** Association between dietary fiber intake and hs-CRP (continuous and elevated) in U.S. adults, NHANES 2017–2018.

Model	% Change in hs-CRP per +5 g/Day (95% CI)	*p*-Value	OR for Elevated hs-CRP † per +5 g/Day (95% CI)	*p*-Value
Model 1: Demographics only (age, sex, race/ethnicity)	−6.99% (−8.61, −5.38)	**<0.001**	0.86 (0.81, 0.91)	**<0.001**
Model 2: + SES, BMI, lifestyle (PIR, smoking, vigorous PA)	−4.67% (−6.11, −3.22)	**<0.001**	0.88 (0.81, 0.94)	**<0.001**
Model 3: + clinical covariates (diabetes, hypertension, hyperlipidemia), energy intake, alcohol category	−4.53% (−6.32, −2.74)	**<0.001**	0.87 (0.81, 0.94)	**<0.001**

Survey-weighted models account for NHANES strata, PSUs, and 2-day dietary weights. Percent change estimates are from linear regressions of log-transformed hs-CRP, transformed as 100 × (exp(β) − 1) using nlcom. Logistic models estimate odds ratios per +5 g/day of fiber. Bolded estimates indicate statistical significance at *p* < 0.05. † Elevated hs-CRP defined as 3.0–9.9 mg/L. Abbreviations: BMI, body mass index; CI, confidence interval; hs-CRP, high-sensitivity C-reactive protein; NHANES, National Health and Nutrition Examination Survey; OR, odds ratio; PA, physical activity; PIR, poverty–income ratio; SES, socioeconomic status.

**Table 3:** Association Between Dietary Fiber Intake Quartiles and hs-CRP in U.S. Adults, NHANES 2017–2018.

Quartile of Fiber Intake	% Difference in ln(hs-CRP) (95% CI)	*p*-Value	Odds Ratio for Elevated hs-CRP (95% CI)	*p*-Value
Q1 (lowest, ref)	Ref	–	Ref	–
Q2	−2.9% (−14.0, 8.2)	0.61	0.93 (0.64, 1.34)	0.67
Q3	−4.7% (−18.4, 8.9)	0.50	0.86 (0.53, 1.40)	0.52
Q4 (highest)	−20.7% (−27.1, −14.4)	**<0.001**	0.53 (0.32, 0.85)	**0.012**
***p*** **-trend**	−7.0% (−10.5, −3.6)	**0.001**	0.83 (0.70, 0.98)	**0.032**
**Pairwise comparisons**				
Q4 vs. Q2	−18.4% (−26.7, −10.1)	**<0.001**	0.57 (0.35, 0.90)	**0.020**
Q4 vs. Q3	−16.8% (−25.6, −8.0)	**<0.001**	0.61 (0.41, 0.88)	**0.011**
Q3 vs. Q2	−1.9% (−14.6, 10.8)	0.77	0.92 (0.61, 1.41)	0.70

Estimates are from survey-weighted regression models adjusted for age, sex, race/ethnicity, socioeconomic status, BMI, smoking, physical activity, diabetes, hypertension, hyperlipidemia, total energy intake, and alcohol consumption. hs-CRP = high-sensitivity C-reactive protein. Abbreviations: CI = confidence interval; hs-CRP = high-sensitivity C-reactive protein; Ref = reference.

**Table 4:** Association between dietary fiber intake and leukocyte indices in U.S. adults, NHANES 2017–2018.

Outcome	Model	% Change per +5 g/Day (95% CI)	*p*-Value
**WBC**	Model 1: Demographics (age, sex, race/ethnicity)	−1.76% (−2.43, −1.09)	**<0.001**
	Model 2: + BMI, lifestyle (PIR, smoking, vigorous PA)	−0.90% (−1.61, −0.20)	**0.012**
	Model 3: + clinical covariates (diabetes, hypertension, hyperlipidemia), energy intake, alcohol category	−0.98% (−1.84, −0.13)	**0.024**
**Neutrophils**	Model 1: Demographics (age, sex, race/ethnicity)	−2.34% (−3.27, −1.40)	**<0.001**
	Model 2: + BMI, lifestyle (PIR, smoking, vigorous PA)	−1.28% (−2.17, −0.40)	**0.005**
	Model 3: + clinical covariates (diabetes, hypertension, hyperlipidemia), energy intake, alcohol category	−1.44% (−2.62, −0.26)	**0.017**

Survey-weighted linear models of log-transformed outcomes; estimates reported as percent change per +5 g/day dietary fiber using 100 × (exp(β) − 1) via nlcom. All models include NHANES strata, PSUs, and 2-day dietary weights. Bold indicates *p* < 0.05. Abbreviations: BMI, body mass index; CI, confidence interval; NHANES, National Health and Nutrition Examination Survey; PA, physical activity; PIR, poverty–income ratio; WBC, white blood cell count.

**Table 5:** Sensitivity analyses of the association between dietary fiber intake and inflammatory biomarkers (NHANES 2017–2018).

Sensitivity Analysis	Sample Size (n)	Outcome	% Change per 5 g/Day (95% CI)	OR for Elevated hs-CRP (95% CI)	Secondary Outcomes (WBC, Neutrophils) *
**S1**. Healthy subset	640	ln(hs-CRP)	−4.6% (−9.9, 0.7)	0.91 (0.70−1.16)	WBC: −1.0% (−2.1, 0.2); Neut: −1.7% (−3.7, 0.3)
**S2**. Exclude adiposity covariates	3083	ln(hs-CRP)	**−6.3% (−8.1, −4.4)**	**0.85 (0.80−0.91)**	**WBC: −1.2% (−2.1, −0.4); Neut: −1.8% (−2.9, −0.6)**
**S3A**. Alcohol as continuous grams/day	1833	ln(hs-CRP)	**−6.4% (−9.3, −3.6)**	**0.82 (0.73–0.90)**	WBC: −1.0% (−2.2, 0.3); Neut: −1.0% (−2.7, 0.6)
**S3B.** Exclude heavy drinkers	1607	ln(hs-CRP)	**−5.6% (−8.6, −2.6)**	**0.83 (0.74–0.93)**	WBC: −0.9% (−2.3, 0.5); Neut: −0.7% (−2.6, 1.3)

* Secondary outcomes are expressed as percent change in WBC or neutrophil count per 5 g/day increment of dietary fiber intake, with 95% confidence intervals. All models are survey-weighted linear or logistic regressions using NHANES design variables. Models adjust for age, sex, race/ethnicity, socioeconomic status, smoking, physical activity, clinical covariates (diabetes, hypertension, hyperlipidemia), total energy intake, and alcohol consumption as specified in each sensitivity analysis. Bolded estimates indicate statistical significance at *p* < 0.05. Abbreviations: CI, confidence interval; hs-CRP, high-sensitivity C-reactive protein; ln, natural logarithm; Neut, neutrophil count; NHANES, National Health and Nutrition Examination Survey; OR, odds ratio; WBC, white blood cell count.

## Data Availability

All data used in this study are publicly available from the National Health and Nutrition Examination Survey (NHANES) 2017–2018 public-use release, including documentation and codebooks, at the U.S. Centers for Disease Control and Prevention (CDC) website. The derived analytic dataset and reproducibility code supporting the findings of this study are available on Zenodo (DOI: 10.5281/zenodo.18757573).

## Supplementary Materials

The following supporting information can be downloaded at: https://www.mdpi.com/article/10.3390/nu18060972/s1, Table S1: Definition, coding, and analytic role of study variables in NHANES 2017–2018; Figure S1: Directed acyclic graph (DAG) illustrating the hypothesized relationships between dietary fiber intake and systemic inflammation in NHANES 2017–2018; Figure S2: Distribution of hs-CRP (mg/L) in the analytic cohort (NHANES 2017–2018); Figure S3: Unadjusted associations between dietary fiber intake and inflammatory markers; Figure S4: Dose–response of dietary fiber with secondary inflammatory cell counts.

## Abbreviations

The following abbreviations are used in this manuscript:

AHA	American Heart Association
BMI	body mass index
CDC	Centers for Disease Control and Prevention
CI	confidence interval
CKD	chronic kidney disease
COPD	chronic obstructive pulmonary disease
COVID-19	coronavirus disease 2019
CRP	C-reactive protein
CVD	cardiovascular disease
DAS28-CRP	Disease Activity Score in 28 joints using C-reactive protein
DMARDs	disease-modifying antirheumatic drugs
eGFR	estimated glomerular filtration rate
GPR	G protein-coupled receptor
HCA2	hydroxycarboxylic acid receptor 2
HDL	high-density lipoprotein
hs-CRP	high-sensitivity C-reactive protein
IL	interleukin
IQR	interquartile range
LDL	low-density lipoprotein
ln	natural logarithm
Neut	neutrophil count
NHANES	National Health and Nutrition Examination Survey
NSAIDs	nonsteroidal anti-inflammatory drugs
OR	odds ratio
PA	physical activity
PIR	poverty–income ratio
PSUs	primary sampling units
RA	rheumatoid arthritis
RCS	restricted cubic splines
RCTs	randomized controlled trials
Ref	reference
SCFAs	short-chain fatty acids
SES	socioeconomic status
Th17	T helper 17 cells
TNF-α	tumor necrosis factor-alpha
TNF-R2	tumor necrosis factor-alpha receptor 2
WBC	white blood cell count

## References

[1] Pearson T.A., Mensah G.A., Alexander R.W., Anderson J.L., Cannon R.O., Criqui M., Fadl Y.Y., Fortmann S.P., Hong Y., Myers G.L. (2003). Markers of Inflammation and Cardiovascular Disease: Application to Clinical and Public Health Practice: A Statement for Healthcare Professionals From the Centers for Disease Control and Prevention and the American Heart Association. Circulation.

[2] Fatima Z.M., Shakeel R.M., Chaudhry S.A.A.M., Qadri M.M., Kakar A.I.M., Ahmad B.M., Ahmad T.M., Akilimali A.B. (2025). The relation between C-reactive protein (CRP) and risk of incident heart failure in patients with cardiovascular disease: A narrative review. Ann. Med. Surg..

[3] Hu B., Liu T., Sun Y., Sun J., Feng L., Li F. (2025). Association between systemic immune-inflammation index and all-cause and CVD mortality in non-elderly diabetic adults. Clinics.

[4] Li T., Yu Q., Wang Y., Cai X., Kong Y., Zhao H., Diao S., Qin Y., Fang Q. (2022). High-sensitivity C-reactive protein as a better predictor of post-thrombolytic functional outcome in patients with previous antiplatelet therapy. Eur. J. Med. Res..

[5] Santas E., Villar S., Palau P., Llàcer P., de la Espriella R., Miñana G., Lorenzo M., Núñez-Marín G., Górriz J.L., Carratalá A. (2024). High-sensitivity C-reactive protein and risk of clinical outcomes in patients with acute heart failure. Sci. Rep..

[6] Furman D., Campisi J., Verdin E., Carrera-Bastos P., Targ S., Franceschi C., Ferrucci L., Gilroy D.W., Fasano A., Miller G.W. (2019). Chronic inflammation in the etiology of disease across the life span. Nat. Med..

[7] Fonseca F.A.H., Izar M.C.d.O. (2016). High-Sensitivity C-Reactive Protein and Cardiovascular Disease Across Countries and Ethnicities. Clinics.

[8] Mehta A., Blumenthal R.S., Gluckman T.J., Feldman D.I., Kohli P. (2025). High-sensitivity C-reactive Protein in Atherosclerotic Cardiovascular Disease: To Measure or Not to Measure?. US Cardiol. Rev..

[9] Romero-Cabrera J.L., Ankeny J., Fernández-Montero A., Kales S.N., Smith D.L. (2022). A Systematic Review and Meta-Analysis of Advanced Biomarkers for Predicting Incident Cardiovascular Disease among Asymptomatic Middle-Aged Adults. Int. J. Mol. Sci..

[10] Mainous A.G., Sharma P., Jo A. (2023). Systemic inflammation among adults with diagnosed and undiagnosed cardiometabolic conditions: A potential missed opportunity for cardiovascular disease prevention. Front. Med..

[11] Liu C., Li C. (2023). C-reactive protein and cardiovascular diseases: A synthesis of studies based on different designs. Eur. J. Prev. Cardiol..

[12] Jin X., Qiu T., Li L., Yu R., Chen X., Li C., Proud C.G., Jiang T. (2023). Pathophysiology of obesity and its associated diseases. Acta Pharm. Sin. B.

[13] Fu L., Zhang G., Qian S., Zhang Q., Tan M. (2022). Associations between dietary fiber intake and cardiovascular risk factors: An umbrella review of meta-analyses of randomized controlled trials. Front. Nutr..

[14] Benedicto-Toboso M.I., Salviano A.F., Miguel-Berges M.L., Torre I.R.-D., Moreno L.A., Santaliestra-Pasías A.M. (2025). Effect of Dietary Fiber Intake on Chronic Low-Grade Inflammation in Children and Adolescents: A Systematic Review and Meta-analysis of Randomized Controlled Trials. Curr. Dev. Nutr..

[15] Reyneke G.L., Lambert K., Beck E.J. (2025). Dietary Patterns Associated with Anti-inflammatory Effects: An Umbrella Review of Systematic Reviews and Meta-analyses. Nutr. Rev..

[16] Smolen J.S., Breedveld F.C., Burmester G.R., Bykerk V.P., Dougados M., Emery P., Kvien T.K., Navarro-Compán M.V., Oliver S., Schoels M. (2016). Treating rheumatoid arthritis to target: 2014 update of the recommendations of an international task force. Ann. Rheum. Dis..

[17] Zhang D., Jian Y.-P., Zhang Y.-N., Li Y., Gu L.-T., Sun H.-H., Liu M.-D., Zhou H.-L., Wang Y.-S., Xu Z.-X. (2023). Short-chain fatty acids in diseases. Cell Commun. Signal..

[18] Kupczyk D., Bilski R., Szeleszczuk Ł., Mądra-Gackowska K., Studzińska R. (2025). The Role of Diet in Modulating Inflammation and Oxidative Stress in Rheumatoid Arthritis, Ankylosing Spondylitis, and Psoriatic Arthritis. Nutrients.

[19] Blank R.B., Bu K., Zhang X., Chen W., Cunningham I., Sokolove J., Lahey L., Heguy A., Medina R., Ubeda C. (2025). Short-chain fatty acids and their gut microbial pathways distinguish rheumatoid arthritis in discordant monozygotic twins. Ann. Rheum. Dis..

[20] Liu Y., Guan S., Xu H., Zhang N., Huang M., Liu Z. (2023). Inflammation biomarkers are associated with the incidence of cardiovascular disease: A meta-analysis. Front. Cardiovasc. Med..

[21] Ale L., Gentleman R., Sonmez T.F., Sarkar D., Endres C. (2024). nhanesA: Achieving transparency and reproducibility in NHANES research. Database.

[22] Chen T.-C., Clark J., Riddles M.K., Mohadjer L.K., Fakhouri T.H. (2020). National Health and Nutrition Examination Survey, 2015-2018: Sample Design and Estimation Procedures. Vital Health Stat..

[23] Paulose-Ram R., Graber J.E., Woodwell D., Ahluwalia N. (2021). The National Health and Nutrition Examination Survey (NHANES), 2021–2022: Adapting Data Collection in a COVID-19 Environment. Am. J. Public Health.

[24] Ismail L.C., Osaili T.M., Mohamad M.N., Al Marzouqi A., Habib-Mourad C., Abu Jamous D.O., Ali H.I., Al Sabbah H., Hasan H., Hassan H. (2022). Assessment of Dietary and Lifestyle Responses After COVID-19 Vaccine Availability in Selected Arab Countries. Front. Nutr..

[25] Rogers A.M., Lauren B.N., Baidal J.A.W., Ozanne E.M., Hur C. (2021). Persistent effects of the COVID-19 pandemic on diet, exercise, risk for food insecurity, and quality of life: A longitudinal study among U.S. adults. Appetite.

[26] Bryan S., Afful J., Carroll M.D., Te-Ching C., Orlando D., Fink S., Fryar C.D., Stierman B., Chen T.-C., Davy O. (2021). National Health and Nutrition Examination Survey 2017–March 2020 Pre-pandemic Data Files—Development of Files and Prevalence Estimates for Selected Health Outcomes.

[27] Albiña-Palmarola P. (2026). NHANES 2017–2018 Dietary Fiber and Inflammation Analysis Dataset and Code. 1.0 Ed, Zenodo. https://zenodo.org/records/18757573.

[28] Meyers G.R., Samouda H., Bohn T. (2022). Short Chain Fatty Acid Metabolism in Relation to Gut Microbiota and Genetic Variability. Nutrients.

[29] Wang L., He L., Xu L., Li S. (2024). Short-chain fatty acids: Bridges between diet, gut microbiota, and health. J. Gastroenterol. Hepatol..

[30] Ney L.-M., Wipplinger M., Grossmann M., Engert N., Wegner V.D., Mosig A.S. (2023). Short chain fatty acids: Key regulators of the local and systemic immune response in inflammatory diseases and infections. Open Biol..

[31] Xie L., Alam J., Marques F.Z., Mackay C.R. (2023). A major mechanism for immunomodulation: Dietary fibres and acid metabolites. Semin. Immunol..

[32] Reynolds A.N., Akerman A., Kumar S., Pham H.T.D., Coffey S., Mann J. (2022). Dietary fibre in hypertension and cardiovascular disease management: Systematic review and meta-analyses. BMC Med..

[33] Zhang D., Frenette P.S. (2019). Cross talk between neutrophils and the microbiota. Blood.

[34] Kim C.H. (2023). Complex regulatory effects of gut microbial short-chain fatty acids on immune tolerance and autoimmunity. Cell. Mol. Immunol..

[35] Smith J.A., Colbert R.A. (2014). Review: The Interleukin-23/Interleukin-17 Axis in Spondyloarthritis Pathogenesis: Th17 and Beyond. Arthritis Rheumatol..

[36] McInnes I.B.P., Schett G.P. (2017). Pathogenetic insights from the treatment of rheumatoid arthritis. Lancet.

[37] Shivakoti R., Biggs M.L., Djoussé L., Durda P.J., Kizer J.R., Psaty B., Reiner A.P., Tracy R.P., Siscovick D., Mukamal K.J. (2022). Intake and Sources of Dietary Fiber, Inflammation, and Cardiovascular Disease in Older US Adults. JAMA Netw. Open.

[38] Ma Y., Griffith J.A., Chasan-Taber L., Olendzki B.C., Jackson E., Stanek E.J., Li W., Pagoto S.L., Hafner A.R., Ockene I.S. (2006). Association between dietary fiber and serum C-reactive protein. Am. J. Clin. Nutr..

[39] Qi L., van Dam R.M., Liu S., Franz M., Mantzoros C., Hu F.B. (2006). Whole-Grain, Bran, and Cereal Fiber Intakes and Markers of Systemic Inflammation in Diabetic Women. Diabetes Care.

[40] Nettleton J.A., Steffen L.M., Mayer-Davis E.J., Jenny N.S., Jiang R., Herrington D.M., Jacobs D.R. (2006). Dietary patterns are associated with biochemical markers of inflammation and endothelial activation in the Multi-Ethnic Study of Atherosclerosis (MESA). Am. J. Clin. Nutr..

[41] Ajani U.A., Ford E.S., Mokdad A.H. (2004). Dietary Fiber and C-Reactive Protein: Findings from National Health and Nutrition Examination Survey Data. J. Nutr..

[42] King D.E., Egan B.M., E Geesey M. (2003). Relation of dietary fat and fiber to elevation of C-reactive protein. Am. J. Cardiol..

[43] Qi X., Li Y., Fang C., Jia Y., Chen M., Chen X., Jia J. (2023). The associations between dietary fibers intake and systemic immune and inflammatory biomarkers, a multi-cycle study of NHANES 2015–2020. Front. Nutr..

[44] Randall Z.D., Brouillard A.M., Deych E., Rich M.W. (2022). Demographic, behavioral, dietary, and clinical predictors of high-sensitivity C-reactive protein: The National Health and Nutrition Examination Surveys (NHANES). Am. Heart J. Plus Cardiol. Res. Pract..

[45] Rhodes D.G., Morton S., Myrowitz R., Moshfegh A.J. (2023). Food and Nutrient Database for Dietary Studies 2019–2020: An application database for national dietary surveillance. J. Food Compos. Anal..

[46] Casagrande S.S., Lawrence J.M. (2024). Cardiovascular disease risk factors and their associations with inflammation among US adolescents: NHANES, 2015 to March 2020. BMJ Open Diabetes Res. Care.

[47] Xie L.-M., Ge Y.-Y., Huang X., Zhang Y.-Q., Li J.-X. (2015). Effects of fermentable dietary fiber supplementation on oxidative and inflammatory status in hemodialysis patients. Int. J. Clin. Exp. Med..

[48] Centers for Disease Control and Prevention (2021). Impact of the COVID-19 Pandemic on Major HHS Data Systems.

[49] U.S. Department of Agriculture and U.S. Department of Health and Human Services (2020). Dietary Guidelines for Americans, 2020–2025.

[50] Dahl W.J., Stewart M.L. (2015). Position of the Academy of Nutrition and Dietetics: Health Implications of Dietary Fiber. J. Acad. Nutr. Diet..

[51] Papandreou P., Gioxari A., Daskalou E., Grammatikopoulou M.G., Skouroliakou M., Bogdanos D.P. (2023). Mediterranean Diet and Physical Activity Nudges versus Usual Care in Women with Rheumatoid Arthritis: Results from the MADEIRA Randomized Controlled Trial. Nutrients.

[52] Liu L., Xie S. (2023). Dietary fiber intake associated with risk of rheumatoid arthritis among U.S. adults: NHANES 2010-2020. Medicine.

[53] Wan H., Zhang Y., Ning Z., Liu M., Yang S. (2024). Associations of cereal fiber intake with rheumatoid arthritis mediated by dietary inflammatory index: Insights from NHANES 2011–2020. Sci. Rep..

[54] Conway J.M., Ingwersen L.A., Moshfegh A.J. (2004). Accuracy of dietary recall using the USDA five-step multiple-pass method in men: An observational validation study. J. Am. Diet. Assoc..

[55] Freedman L.S., Commins J.M., Moler J.E., Arab L., Baer D.J., Kipnis V., Midthune D., Moshfegh A.J., Neuhouser M.L., Prentice R.L. (2014). Pooled Results From 5 Validation Studies of Dietary Self-Report Instruments Using Recovery Biomarkers for Energy and Protein Intake. Am. J. Epidemiol..

[56] Subar A.F., Freedman L.S., Tooze J.A., Kirkpatrick S.I., Boushey C., Neuhouser M.L., Thompson F.E., Potischman N., Guenther P.M., Tarasuk V. (2015). Addressing Current Criticism Regarding the Value of Self-Report Dietary Data. J. Nutr..

[57] Archer E., Hand G.A., Blair S.N. (2013). Validity of U.S. Nutritional Surveillance: National Health and Nutrition Examination Survey Caloric Energy Intake Data, 1971–2010. PLoS ONE.

[58] Ockene I.S., Matthews C.E., Rifai N., Ridker P.M., Reed G., Stanek E. (2001). Variability and Classification Accuracy of Serial High-Sensitivity C-Reactive Protein Measurements in Healthy Adults. Clin. Chem..

[59] Gough A., Sitch A., Ferris E., Marshall T. (2024). Within-subject variation of C-reactive protein and high-sensitivity C-reactive protein: A systematic review and meta-analysis. PLoS ONE.

[60] Holscher H.D. (2017). Dietary fiber and prebiotics and the gastrointestinal microbiota. Gut Microbes.

[61] Hedrick V.E., Savla J., Comber D.L., Flack K.D., Estabrooks P.A., Nsiah-Kumi P.A., Ortmeier S., Davy B.M. (2012). Development of a Brief Questionnaire to Assess Habitual Beverage Intake (BEVQ-15): Sugar-Sweetened Beverages and Total Beverage Energy Intake. J. Acad. Nutr. Diet..

