# Supplementary material for: Association Between Dietary Fiber Intake and Inflammatory Biomarkers in U.S. Adults: A Cross-Sectional Analysis of the Pre-COVID-19 National Health and Nutrition Examination Survey 2017–2018
Source: Nutrients. 2026 Mar 19;18(6):972. doi: 10.3390/nu18060972 (PMC13029420; doi:10.3390/nu18060972)
Supplement: Supplementary file 1 [file nutrients-18-00972-s001.zip › nutrients-4210927-supplementary.pdf]

Supplementary Material

Association Between Dietary Fiber Intake and Systemic Inflammation in U.S. Adults Using NHANES 2017–2018, a Pre-COVID-19 Cycle

1. Supplementary Methods. Additional Variable Construction and Sensitivity Analyses

This section provides detailed operational definitions and coding procedures for selected covariates, including alcohol intake, hyperlipidemia, kidney function, central adiposity, and medication use, which complement the summary information shown in Supplementary Table S1.

1.1. Alcohol consumption

Alcohol intake was derived from the NHANES Alcohol Use Questionnaire (ALQ) for the 2017–2018 cycle. Participants reported the frequency of drinking in the past 12 months (ALQ121) and the average number of drinks consumed on drinking days (ALQ130). Frequency categories were converted into approximate drinking days per year (e.g., 365 for "every day," 104 for "2 times per week," 5 for "3–6 times per year") based on the NHANES codebook. These values were multiplied by the reported number of drinks per drinking day to calculate annual totals, which were then divided by 365 to obtain average drinks per day. This value was multiplied by 14 g (standard ethanol content of one U.S. drink) to estimate grams of ethanol consumed per day. Alcohol consumption was categorized into sex-specific groups based on U.S. dietary guidelines: none (0 g/day), light–moderate (>0–14 g/day for women; >0–28 g/day for men), and heavy (>14 g/day for women; >28 g/day for men). This categorical variable was included as a lifestyle covariate in fully adjusted models. In sensitivity analyses, alcohol intake was modeled continuously (grams/day) using restricted cubic splines to account for potential nonlinearity.

1.2. Hyperlipidemia

Hyperlipidemia was defined as meeting at least one of the following criteria: total cholesterol ≥200 mg/dL, LDL cholesterol ≥130 mg/dL, HDL cholesterol <40 mg/dL in men or <50 mg/dL in women, triglycerides ≥150 mg/dL, self-reported physician diagnosis of high cholesterol, or use of lipid-lowering medications. Lipid-lowering medication use was ascertained from the NHANES prescription medications file (RXQ_RX). Hyperlipidemia was included as a binary covariate in fully adjusted models.

1.3. Kidney function

Kidney function was estimated using the CKD-EPI 2021 race-free equation based on serum creatinine (LBXSCR), age, and sex. Estimated glomerular filtration rate (eGFR, mL/min/1.73 m^2) was calculated for all participants with valid data. Chronic kidney disease (CKD) was defined as eGFR <60 mL/min/1.73 m^2. eGFR was included as a continuous covariate in robustness analyses, and additional sensitivity analyses were performed excluding participants with CKD.

1.4. Central adiposity

Waist circumference (BMXWAIST, cm) was measured at the level just above the iliac crest. A binary variable for central obesity was created using sex-specific NIH cutoffs (≥102 cm in men, ≥88 cm in women). Central obesity was considered in sensitivity

analyses as an alternative to body mass index (BMI) to evaluate robustness to different measures of adiposity.

1.5. Medication use

Medication use was assessed from the NHANES prescription medications file (RXQ_RX). Binary indicators were created for systemic non-aspirin nonsteroidal anti-inflammatory drugs (NSAIDs; e.g., ibuprofen, naproxen, diclofenac, meloxicam, celecoxib), systemic corticosteroids (e.g., prednisone, methylprednisolone, dexamethasone, hydrocortisone), and immunosuppressant/DMARDs (e.g., methotrexate, azathioprine, cyclosporine, tacrolimus, mycophenolate, hydroxychloroquine). Statins were not modeled separately, since they were already incorporated into the hyperlipidemia composite variable. Over-the-counter NSAID use not captured by RXQ_RX is a limitation.

2. Supplementary Tables and Figures

Table S1. Definition, coding, and analytic role of study variables in NHANES 2017–2018

Variable	NHANES Code(s)	Role	Rationale
Dietary fiber intake	DR1TFIBE, DR2TFIBE	Exposure	Primary dietary exposure; hypothesized to reduce systemic inflammation. Two-day average intake used.
High-sensitivity CRP (hs-CRP)	LBXHSCRP	Outcome	Gold-standard biomarker of systemic low-grade inflammation. Values > 10 mg/L excluded to minimize influence of acute infection.
White blood cell count	LBXWBCSI	Outcome	General immune activation marker.
Neutrophil count	Derived: (LBXNEPCT/100) × LBXWBCSI	Outcome	Reflects innate immune activity; sensitive to systemic inflammation. Derived because NHANES does not provide absolute counts directly.
Age	RIDAGEYR	Confounder	Strongly associated with both dietary intake and inflammation.
Sex	RIAGENDR	Confounder	Biological and behavioral differences in diet and immune function.
Race/Ethnicity	RIDRETH3	Confounder	Social determinant influencing diet and inflammatory status.
Education	DMDEDUC2 (collapsed: ≤High school, Some college/AA, College graduate+)	Confounder	Proxy for socioeconomic status; linked to dietary patterns and health behaviors.
Family Poverty–Income Ratio (PIR)	INDFMPIR	Confounder	Standard socioeconomic indicator; affects access to healthy foods and risk of inflammation.
Total energy intake	DR1TKCAL, DR2TKCAL	Confounder	Controls for overall diet quantity; distinguishes fiber intake from total caloric intake.
Smoking status	SMQ020 (≥100 cigarettes in lifetime)	Confounder	Pro-inflammatory exposure, also influences diet quality.
Physical activity (vigorous)	PAQ605	Confounder	Health behavior with anti-inflammatory effects; associated with diet quality.
Alcohol consumption category	ALQ130 (drinks/day), ALQ120Q/ALQ120U (frequency), sex-specific thresholds	Confounder	Categorized as none, light–moderate, or heavy drinking based on sex-specific U.S. guidelines; alcohol is linked to both diet and inflammation.
Hyperlipidemia	Composite: LBXTC, LBDLDL, LBXTR, HDL (LBDHDD/LBDHDL + sex-specific cutoffs), BPQ080, lipid-lowering medications (RXQ_RX_J + RXQ_DRUG)	Confounder	Present if any: total cholesterol ≥ 200 mg/dL, LDL ≥ 130 mg/dL, HDL < 40 mg/dL (men) or < 50 mg/dL (women), triglycerides ≥ 150 mg/dL, self-reported diagnosis of high cholesterol, or use of lipid-lowering medications.
Body mass index (BMI)	BMXBMI	Potential mediator	Fiber may reduce adiposity, which in turn lowers inflammation.

Variable	NHANES Code(s)	Role	Rationale
Central obesity	BMXWAIST (waist ≥102 cm men, ≥88 cm women)	Potential mediator	Central fat distribution is metabolically active and closely related to systemic inflammation.
Hypertension	BPQ020	Potential mediator	Chronic condition influenced by diet, linked to inflammation.
Diabetes	DIQ010	Potential mediator	Diet improves metabolic health and reduces inflammation.
Chronic kidney disease (CKD)	LBXSCR, RIDAGEYR, RIAGENDR → CKD-EPI 2021 formula	Potential mediator	Reduced kidney function is associated with systemic inflammation and metabolic status.
Other chronic conditions	MCQ variables (asthma, arthritis, COPD, CVD, cancer)	Descriptive	Provide health context; potentially related to inflammation but not modeled as confounders.
Medication use	RXQ_RX_J (NSAIDs, corticosteroids, DMARDs)	Descriptive	Captures use of anti-inflammatory drugs; relevant to interpretation but not modeled as confounders.

Table S1 summarizes the operational definitions, NHANES source variables, coding schemes, and analytic roles of all study variables. Variables are grouped into exposures, outcomes, confounders, and potential mediators, consistent with the study's conceptual model. Dietary fiber intake was defined as the two-day average of total fiber from the 24-hour recalls. Dietary fiber quartiles (fiber_avg; g/day) were defined using unweighted cutpoints from the analytic sample (n=3,570): Q1 0.20–10.10, Q2 10.10–15.00, Q3 15.05–21.50, and Q4 21.55–81.90. Systemic inflammation was captured by hs-CRP, white blood cell count, and derived neutrophil counts, with hs-CRP values >10 mg/L excluded to reduce acute infection bias. Sociodemographic and behavioral covariates (age, sex, race/ethnicity, education, family poverty–income ratio, smoking, physical activity, and alcohol use) were selected a priori as confounders based on prior evidence. Alcohol exposure was represented by sex-specific categories of consumption (none, light–moderate, heavy). Hyperlipidemia was constructed as a composite indicator (laboratory, self-report, and medication use) to capture lipid abnormalities comprehensively. Body mass index, central obesity, hypertension, diabetes, and chronic kidney disease were considered potential mediators, reflecting metabolic pathways through which dietary fiber may influence inflammation. Additional chronic conditions and medication use were described for context.

Figure S1. Directed acyclic graph (DAG) illustrating the hypothesized relationships between dietary fiber intake and systemic inflammation in NHANES 2017–2018.

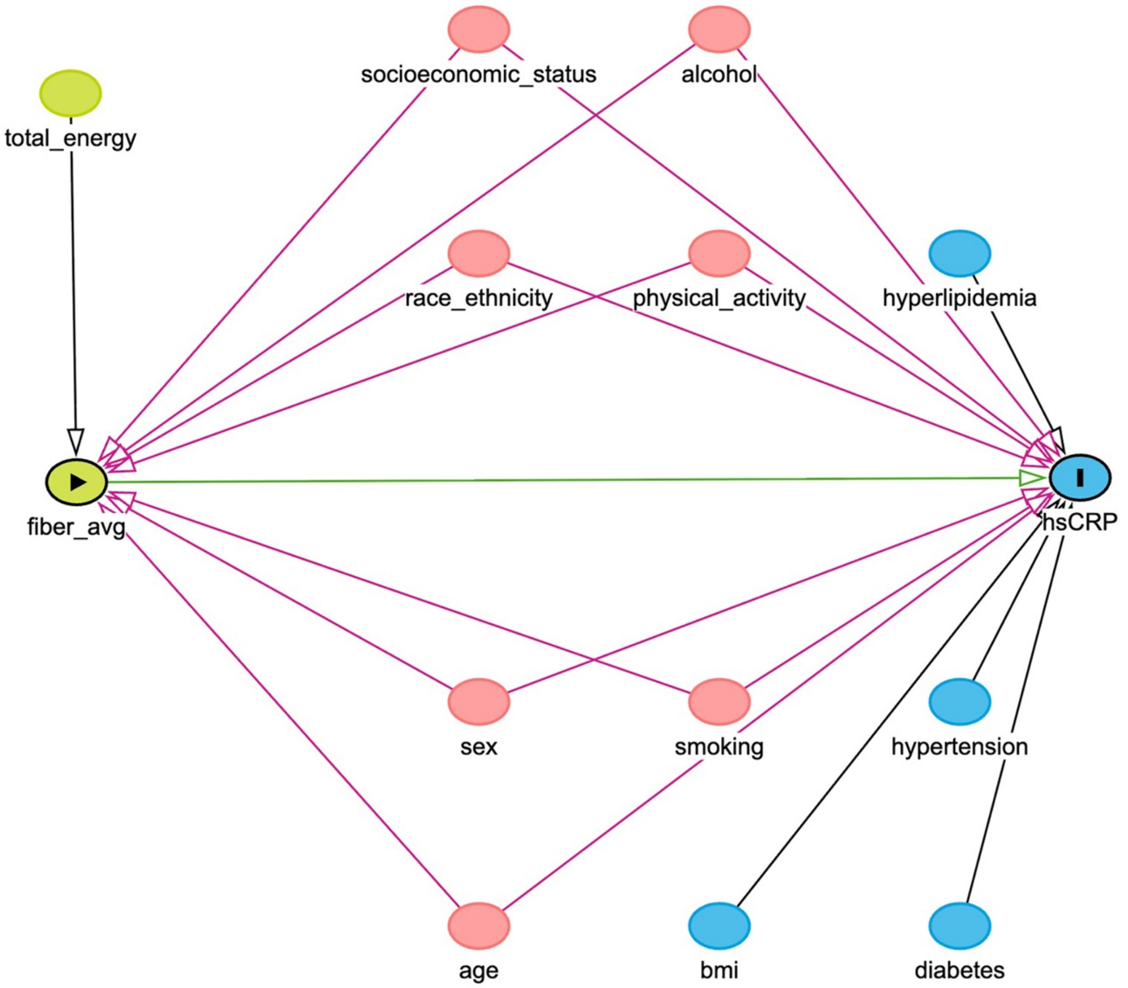

The diagram illustrates the assumed causal structure for the association between dietary fiber intake (fiber_avg; exposure) and serum high-sensitivity C-reactive protein (hs-CRP; outcome). Demographic and socioeconomic factors (age, sex, race/ethnicity, education, and poverty–income ratio) were considered upstream confounders that may influence both dietary fiber intake and inflammatory status. Lifestyle factors (smoking, physical activity, alcohol intake) were modeled as additional potential confounders. Total energy intake was modeled as a determinant of dietary fiber intake. Adiposity and cardiometabolic conditions (BMI/central obesity, hypertension, diabetes, hyperlipidemia) may lie on the causal pathway (potential mediators) and are also strong determinants of inflammation; these were examined in sequential models and sensitivity analyses to assess robustness to alternative adjustment strategies. Arrows represent hypothesized causal directions. Legend: Exposure: dietary fiber intake (fiber_avg). Outcome: hs-CRP. Variables with arrows into both exposure and outcome (age, sex, race/ethnicity, socioeconomic status, alcohol intake, smoking, physical activity) were treated as potential confounders. Variables with arrows into hs-CRP only (BMI, diabetes, hypertension, hyperlipidemia) were considered outcome predictors and potential mediators examined in sequential models/sensitivity analyses. Total energy intake was modeled as a determinant of fiber intake.

Figure S2. Distribution of hs-CRP (mg/L) in the analytic cohort (NHANES 2017–2018).

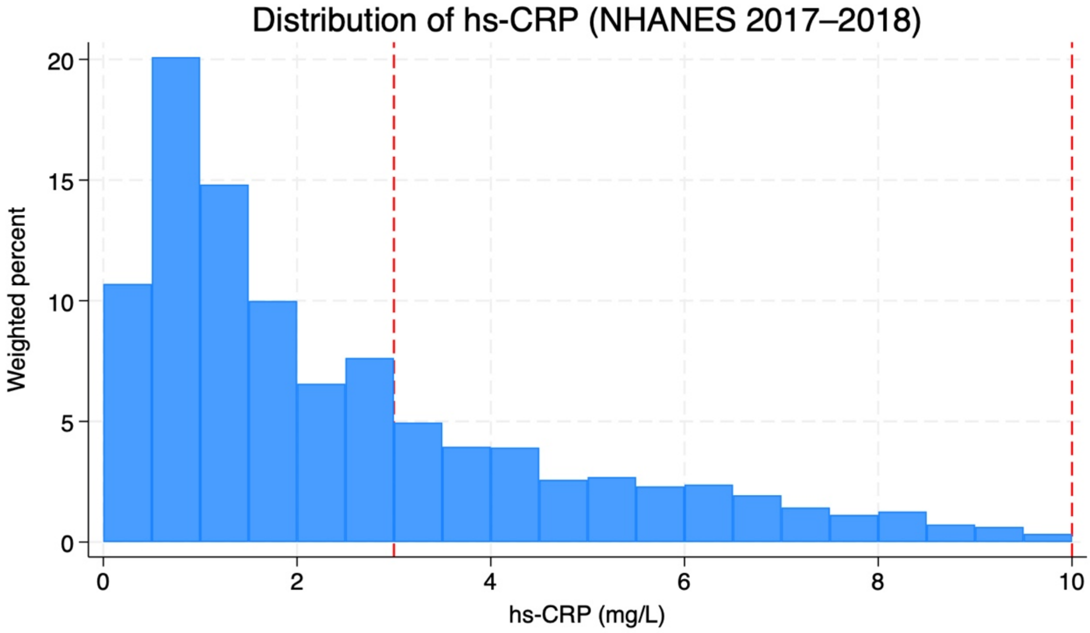

Weighted histogram (bin width 0.5 mg/L) of serum high-sensitivity C-reactive protein (hs-CRP) concentrations among adults included in the analytic sample (restricted to hs-CRP ≤10 mg/L). Vertical dashed lines mark the analytic cutoffs at 3 mg/L (primary stratification threshold) and 10 mg/L (exclusion threshold). Percentages are survey-weighted to account for the NHANES complex sampling design.

Figure S3. Unadjusted associations between dietary fiber intake and inflammatory markers.

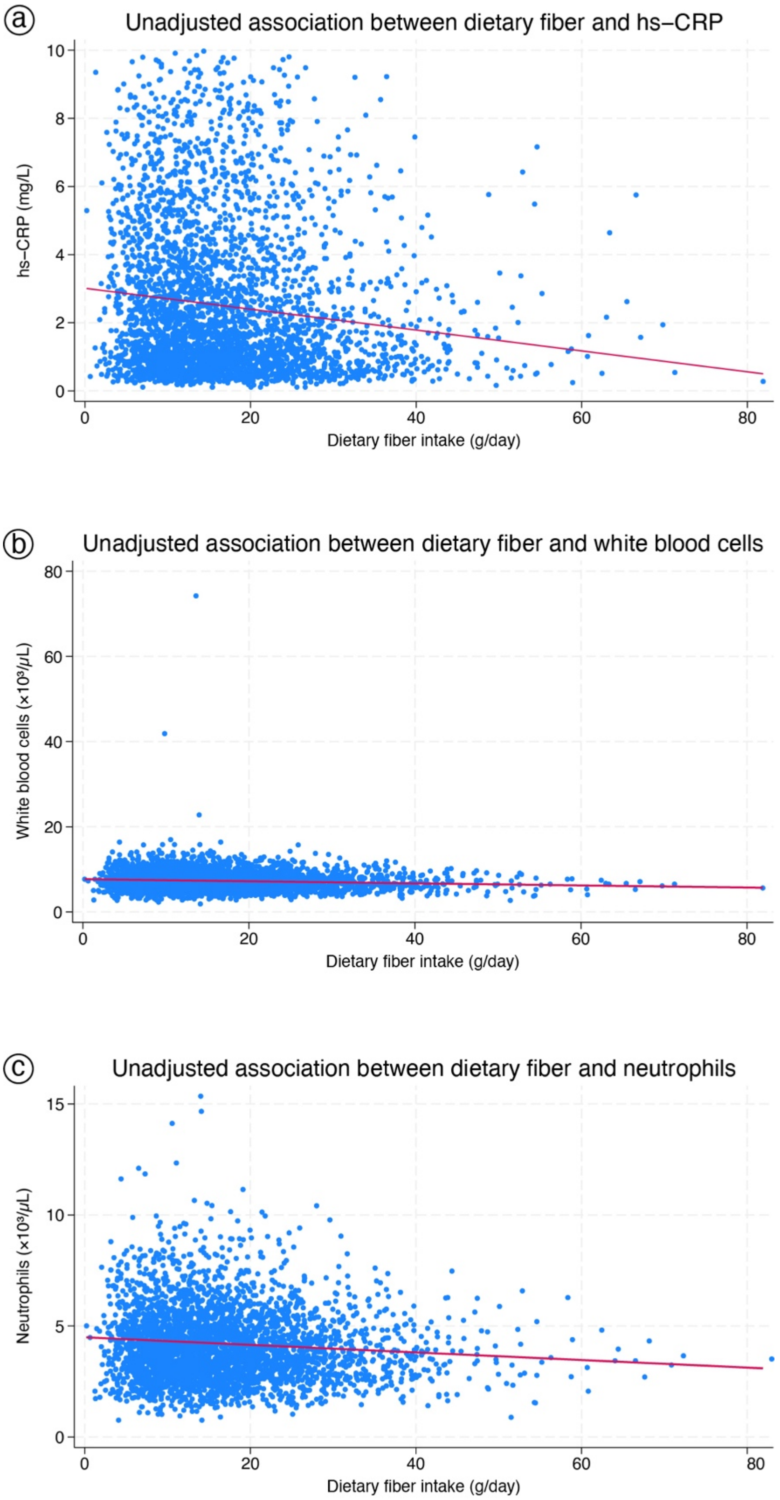

Scatter plot of dietary fiber intake (*g/day*) versus serum hs-CRP (*mg/L*) **(A)**, versus white blood cell count (WBC, ×10³/*μL*) **(B)**, and versus neutrophil count (×10³/*μL*) **(C)**. All plots are based on unadjusted, survey-weighted linear regressions. Raw data points are shown with superimposed weighted linear fit lines. Abbreviations: hs-CRP, high-sensitivity C-reactive protein; NHANES, National Health and Nutrition Examination Survey; WBC, white blood cells.

Figure S4. Dose–response of dietary fiber with secondary inflammatory cell counts.

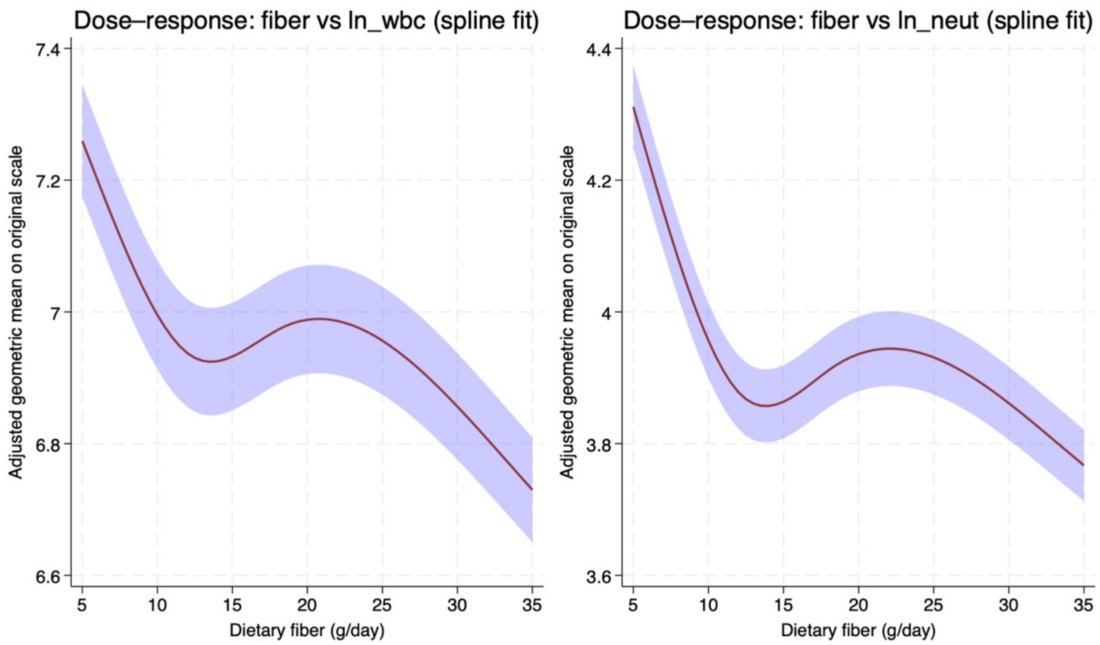

Survey-weighted restricted cubic spline models (knots at the 5th, 35th, 65th, and 95th percentiles of fiber) show adjusted geometric means (solid lines) and 95% CIs (shaded bands) for (A) total leukocyte count (WBC) and (B) neutrophil count across 5–35 g/day fiber. Models adjust for age, sex, race/ethnicity, income-to-poverty ratio, BMI, smoking, vigorous physical activity, diabetes, hypertension, hyperlipidemia, total energy intake, and alcohol category. Non-linearity tests: WBC p=0.227; neutrophils p=0.0017.
